# Trajectory of the main GABAergic interneuron populations from early development to old age in the rat primary auditory cortex

**DOI:** 10.3389/fnana.2014.00040

**Published:** 2014-06-02

**Authors:** Lydia Ouellet, Etienne de Villers-Sidani

**Affiliations:** Department of Neurology and Neurosurgery, Montreal Neurological InstituteMontreal, QC, Canada

**Keywords:** aging, auditory, A1, inhibition, GABA, parvalbumin, somatostatin, interneurons

## Abstract

In both humans and rodents, decline in cognitive function is a hallmark of the aging process; the basis for this decrease has yet to be fully characterized. However, using aged rodent models, deficits in auditory processing have been associated with significant decreases in inhibitory signaling attributed to a loss of GABAergic interneurons. Not only are these interneurons crucial for pattern detection and other large-scale population dynamics, but they have also been linked to mechanisms mediating plasticity and learning, making them a prime candidate for study and modeling of modifications to cortical communication pathways in neurodegenerative diseases. Using the rat primary auditory cortex (A1) as a model, we probed the known markers of GABAergic interneurons with immunohistological methods, using antibodies against gamma aminobutyric acid (GABA), parvalbumin (PV), somatostatin (SOM), calretinin (CR), vasoactive intestinal peptide (VIP), choline acetyltransferase (ChAT), neuropeptide Y (NPY), and cholecystokinin (CCK) to document the changes observed in interneuron populations across the rat's lifespan. This analysis provided strong evidence that several but not all GABAergic neurons were affected by the aging process, showing most dramatic changes in expression of parvalbumin (PV) and somatostatin (SOM) expression. With this evidence, we show how understanding these trajectories of cell counts may be factored into a simple model to quantify changes in inhibitory signaling across the course of life, which may be applied as a framework for creating more advanced simulations of interneuronal implication in normal cerebral processing, normal aging, or pathological processes.

## Introduction

The cerebral cortex is a complex computational machine, simultaneously processing environmental information through a delicate balance of excitation and inhibition. In the mammalian cortex, this is accomplished by two main neuron subpopulations: excitatory glutamatergic neurons, constituting about 80% of all cortical neurons, and inhibitory GABAergic interneurons, representing 20% of the total neurons population (DeFelipe, 1993; Martinez et al., 2002; Markram et al., 2004; Ascoli et al., 2008; Lehmann et al., 2012). Although inhibitory interneurons constitute a clear minority compared to the number of excitatory neurons, they are highly influential, with important roles in cortical maturation, function, plasticity and organization of complex cortical networks underlying a wide range of functions (Lehmann et al., 2012). The majority of these cells in the cortex are inhibitory, and express gamma-aminobutyric acid (GABA) as their principal neurotransmitter (Dreifuss et al., 1969; Somogyi et al., 1983). Further, they have been shown to have an important role in development and function of the cerebral cortex by acting as a sensory “gate” (Gelman and Marin, 2010; Tong et al., 2013), regulating environmental input, and coordinating the output of multiple projection neurons through attenuating and modulating glutamatergic excitation (Levitt, 2005; Lehmann et al., 2012). Inhibitory interneurons may be divided into multiple subtypes on the basis of morphology, physiology, and biochemistry that serve distinct roles in cortical processing (Markram et al., 2004; Rudy et al., 2011). It is estimated that as many of 20 subtypes exist, representing a broad range of morphology and function (Ascoli et al., 2008). For example, studies performed in rodent brain tissue have suggested that these interneurons in the cerebral cortex are typically identified by either characteristic parvalbumin (PV) or somatostatin (SOM) expression (Gonchar and Burkhalter, 1997; Ascoli et al., 2008). PV-positive cells have been implicated in initiation of a critical period for cortical plasticity in the visual, somatosensory and auditory cortices (Hensch et al., 1998; Fagiolini and Hensch, 2000; Hensch, 2004, 2005; Fox and Wong, 2005; Yuste, 2005; Wonders and Anderson, 2006; de Villers-Sidani et al., 2008; Lehmann et al., 2012) and play a critical role in cortical information processing (Tallon-Baudry et al., 1998; Bartos et al., 2007; Cardin et al., 2009; Sohal et al., 2009), whereas SOM-positive cells are believed to plays a role in the acquisition memory and emotional functions (Vecsei and Widerlov, 1990; Schettini, 1991). These two subtypes represent only a fraction of known interneuron subtypes, and of important roles that they underlie in the cortex. While slightly less is known about other populations, it is sure that their functional and morphological diversity provides a complex foundation for cortical information processing, and regulating healthy levels of excitation and inhibition in specific cortical sub-circuits.

The study of how these interneurons contribute to ongoing cortical processing is still in its infancy, yet it is believed that their dysfunction contributes to many neurological and neuropsychiatric diseases (Sanacora et al., 2000; McBain and Fisahn, 2001; Levitt et al., 2004; Marin, 2012) such as schizophrenia (Lewis, 2000; Lewis et al., 2005), Alzheimer's disease (Verret et al., 2012; Hazra et al., 2013), epilepsy (Cossart et al., 2001; Levitt, 2005) or autism spectrum disorder (Rubenstein and Merzenich, 2003). Several authors have suggested that a fundamental vulnerability of cortical GABAergic interneurons lies at the heart of these and other disorders, pushing the study of their inherent computational role (and failure thereof) to the forefront (Akbarian et al., 1995; Benes et al., 1996; Rubenstein and Merzenich, 2003; Dani et al., 2005; Levitt, 2005; Lewis et al., 2005; Maffei et al., 2006; Yizhar et al., 2011). Thus, accumulating evidence appears to reflect the crucial role of properly-mediated excitation and inhibition in cortical processing, and highlights the need for better understanding inhibitory circuits. Furthermore, it is critical to explore and document how a chronic increase or decrease in particular inhibitory interneuron populations may affect these processes. However, the study and quantification of interneuron dynamics in neuropathology is complicated by natural fluctuations in their population over the lifecourse (Lehmann et al., 2012). A large number of studies have documented the distribution and numbers of various GABAergic cortical interneurons at single time points in rodents and primates (Fitzpatrick et al., 1987; Hendry et al., 1987; Beaulieu, 1993; see Rudy et al., 2011 and DeFelipe et al., 2013 for review). The trajectory of various interneuron markers has also been examined extensively during early development and early adulthood, primarily in rodent models (Gonchar et al., 2008; Bartolini et al., 2013). Only a handful of studies have however examined the impact of natural aging on the various cortical interneuron subtypes, most of them focusing on a small fraction of the interneuron pool (Miettinen et al., 1993; Bu et al., 2003; Pugliese et al., 2004; see Lehmann et al., 2012 for a review). As these changes strongly lend themselves to understanding the development of age-related inhibition-related disorders, it is thus critical to develop a full picture of how interneurons' populations naturally fluctuate. For this current research, we have elected to study the primary auditory cortex (A1), which plays a key role in auditory learning, speech perception, auditory attention, and cognitive analysis of sound. Like other neocortices, it consists of a layered network of excitatory cells and inhibitory interneurons (Letinic et al., 2002; Molnár et al., 2006; Linden, 2012). Inhibitory interneurons regulate A1's processing by shaping the spectral tuning (Froemke et al., 2007; Wu et al., 2008), temporal tuning (Schulze and Langner, 1999; Razak and Fuzessery, 2009), and response dynamics of local excitatory neurons (Wehr and Zador, 2003). To best conceptualize these inherent lifecourse dynamics, we have documented the distribution of 7 different markers of GABAergic inhibitory interneurons on healthy Long-Evans rats from 9 days (P9) at five time point during the rats' life until advanced maturity at 25 months (P750, equivalent to ~75 human years based on this strain's longevity). The specific markers we examined include PV, SOM, calretinin (CR), vasoactive intestinal peptide (VIP), neuropeptide-Y (NPY), cholecystokinin (CCK), and choline acetyl transferase (ChAT). Together these markers reveal around 90% of the overall cortical interneuron population (Gonchar et al., 2008; Xu et al., 2010; Rudy et al., 2011). PV, SOM and CR are useful to divide the interneurons in three mostly non-overlapping groups, which can then be further characterized with the additional markers (Kubota et al., 1994; Gonchar and Burkhalter, 1997; Xu et al., 2004; Butt et al., 2005; Miyoshi et al., 2007). Patterns of GABA and neuron-specific Nissl staining were also documented at each time point to determine the trajectories of inhibitory interneurons and cortical neurons as a whole. Using this information, we constructed a simple graphical model, which may be applied as a framework for creating more advanced simulations of interneuronal implication in normal cerebral processing, normal aging, or pathological processes.

## Materials and methods

### Animals

Experiments were performed in auditory cortex (A1) of Long-Evans rats between postnatal day 9 (P9) and P750 (~25 months). All experimental protocols were approved by the Montreal Neurological Institute Animal Care Committee and complied with the guidelines of the Canadian Council on Animal Care.

### Preparation and determination of A1 borders

**Figure 1:**
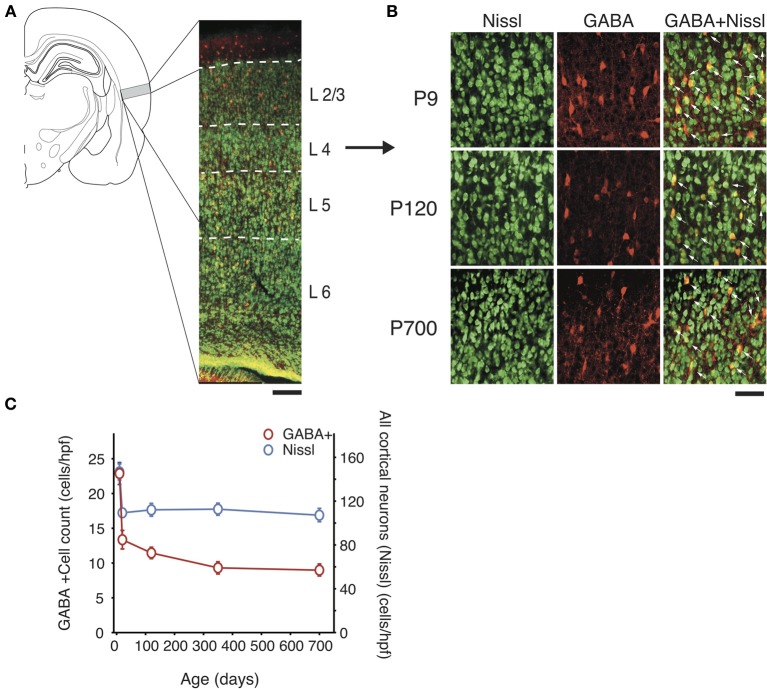
**Total GABAergic cell count and global neuron count across the lifespan in A1. (A)** *Left*, Schematic representation of the location of A1 in the rat brain and, *right*, low power confocal photomicrograph (10X) showing a representative section of the full thickness of A1 (pia up) with cortical layer borders. Neuron-specific Nissl staining is shown in green and GABA staining in red. Scale bar: 100 μm. **(B)** Representative high power micrographs of immunolabeled GABAergic cortical interneurons (red) and Nissl-stained cell bodies (green) and their overlap (yellow with arrow) at three age points. Scale bar: 50 μm. **(C)** GABA immunolabeled cell count and total neural cell count average across all cortical layers over time (*n* = 28 animals with 21 photomicrographs per animal for each marker). Error bars are s.e.m.

All rats were first anaesthetized with pentobarbital (85 mg/kg i.p.) and then perfused through the heart with phosphate buffered saline (pH 7.4, PBS) followed by paraformaldehyde (4%) in 0.1 M PBS. Their brains were removed from the skulls, postfixed in the same fixative overnight and transferred to a 30% sucrose solution, snap-frozen, and stored at −80°C until sectioning. Fixed material was sectioned on a freezing microtome at a 40 μm thickness in the coronal plane along the tonotopic axis of A1. The boundaries of the primary auditory cortex were functionally determined in a subset of animals of different ages with intracortical recordings as previously done (Bao et al., 2003; de Villers-Sidani et al., 2007) using the following criteria: (1) primary auditory neurons generally have a continuous, single-peaked, V-shaped receptive field, and (2) characteristic frequencies of the A1 neurons are tonotopically organized with high frequencies represented rostrally and low frequencies represented caudally. At the end of the recording session, the location of the electrode tracks was converted to coordinates in the Paxinos rat brain atlas. Functional mapping was performed in at least one animal at every age examined except for P9 where tone-evoked responses cannot yet be obtained. For that group, the location of A1 was determined based on mapping done at P12. The cortical borders were defined according to the cell size, density and depth as in (Games and Winer, 1988): layer I (0–175 μm), layers II-III (175–500 μm), layer IV (500–700 μm), and layers V-VI (700–1200 μm). A photomicrograph of the whole cortical thickness showing the border of these cortical layers is shown in Figure 1. For the cell count analysis, 21 photomicrographs were taken in each animal at random locations within the borders of A1 with on average equal sampling in all cortical layers.

### Immunohistochemistry

Sections were treated with PBS 0.1 M 3 × 5 min, followed by mixture of gelatine (2%) and triton X-100 (0.25%) in PBS (PBS-GT) for 4 × 10 min, transferred into primary antibody solution containing PBS-GT and incubated 24–48 h. After incubation, the sections were washed in blocking buffer PBS-GT and incubated for 1 h in dilutions of secondary antibody conjugated with different fluorophores. All primary and secondary antibodies used (see below) were tested for optimal conditions for single and double labeling. The following primary antibodies were used: (1) rabbit anti-gamma amino butyric acid (GABA; Sigma #A2052, 1:5000), (2) mouse anti-PV-19 (Sigma #P3088, 1:10,000), (3) goat anti-CR (Swant #CG1, 1:1000), (4) rat anti-SOM-14 (Chemicon #MAB354, Temecula, CA, 1:1000), (5) rabbit anti-CCK (code L424, a gift from Dr Andrea Varro, Department of physiology, University of Liverpool; see Morino et al., 1994), (6) guinea pig anti-VIP (Peninsula Laboratories #T5030, 1:10,000), (7) guinea pig anti-NPY (Abcam #ab10341, 1:500) (synthetic peptide corresponding to amino acids 76-91 of rat neuropeptide Y), (8) goat anti-ChAT (Chemicon #AB144P, 1:100), and (9) the neuron specific NeuroTrace fluorescent Nissl stain (Molecular Probes #N21480, 1:300). The secondary antibodies were: (1) donkey anti-rabbit (conjugated to Cy3, 1:800, Jackson ImmunoResearch, West Grove, PA; conjugated to AlexaFluor (AF) 488 and 647, 1:800, Jackson) (2) donkey anti-mouse (Cy3, Jackson, 1:800; AF488, Jackson, 1:800; AF647, Invitrogen, 1:800) (3) donkey anti-rat (AF488, Jackson, 1:800) (4) donkey anti-guinea pig (AF488, Jackson, 1:800), and (5) goat anti-rat (AF488, Jackson, 1:800). Stained sections were mounted on 1% gelatin-coated slides, air-dried and cover slipped with mowiol solution (Tris 0.2 M, 30% glycerol, 12% mowiol). Rat tissue from different ages were immunostained in pairs to limit variability related to antibody fixation, incubation time, and postsectioning condition of tissues.

### Microscopy, image acquisition and data analysis

A Zeiss LSM 510 Meta confocal microscope equipped with filter for green Cy2/AF488, red CY3 and infrared CY5/AF647 was used to assess fluorescence in the immunostained sections. To locate A1 in non-functionally mapped animals we used the stereotaxic coordinates (Paxinos): interaural between 5.76 and 2.16 mm and Bregma between −3.24 and −6.84 mm (see above section on determination of A1 borders). To quantify the positive cells, 21 digital images of A1 cortical sections were taken with a 40X objective (Zeiss LSM 510) at random locations within each A1 of each hemisphere for each animal. All quantifications were assessed in 400–500 μm wide A1 sectors (the approximate width of A1 on coronal sections) per hemisphere extending from layer 1 to the underlying white matter. Confocal images were thresholded and adjusted for brightness to maximize the dynamic range of each channel using ImageJ (http://rsb.info.nih.gov/ij/) and adobe Photoshop CS5 (Adobe, San Jose, CA). The goal of our quantitative analyses was to determine the percentage of different subtypes of GABAergic neurons in the auditory cortex (A1) for different age. We determined the number of immune-labeled cells in each section of A1 at different ages (P9, P20, P120, P350, and P700–750) using the optical dissector method (StereoInvestigator software MBF bioscience, Williston, VT) to avoid biased sampling. These counts were then pooled and adjusted to reflect what would have been counted in the whole 40X field. Data were then recorded as an averaged value per high power field (hfp) for each animal and age group. All cells displaying labeling above background levels were counted, regardless of their staining intensity. Data from both hemispheres was pooled. An observer blind to the age of the animal performed all cell counts. Regression analysis was performed in the MatLab environment (Natick, MA) and using the least square methods. Statistical significance between the different age groups and for curve fitting was assessed using ANOVA analysis and the *F*-test. Unless stated otherwise, data are presented as mean ± standard error to the mean (s.e.m).

## Results

### Changes of gabaergic inhibitory interneurons with aging

Our first results using markers for PV, SOM, CR, VIP, ChAT, NPY, and CCK revealed that GABAergic interneuron populations in A1 of rats from 9 to 750 days of age showed considerable fluctuation with time. Surprisingly, we found that even though there was considerable variance between different populations at different time points, the total number of cortical neuron and inhibitory neurons as evidenced by neuron specific Nissl and GABA staining decrease rapidly before postnatal day 20 (P20), by 41 and 77% respectively but afterward remained unchanged (Nissl: from 155 ± 5 to 110 ± 4 cells/hpf; *p* < 0.001; GABA: from 24 ± 3 to 13 ± 2 cells/hpf; *p* < 0.001; Figure 1). Thus, GABA positive cells saw their overall representation in the neuronal pool fall from 16 to 12% over that time period. Since GABA counts remain relatively stable after P20, its relatively small decline later in life cannot account for the global fluctuations in interneuron markers we observed. These results mirror with studies by Pinto et al. (2010) that confirmed that the basal pool of GABA in human is relatively maintained across the lifespan. However, this finding also indicates that some interneuron subtypes may up- or down-regulate expression of certain markers, perhaps compensating for changes in cortical networks resulting from learning or degeneration. Letzkus et al. (2011) have previously suggested this mechanism, in part of a wider mechanism of cortical disinhibition. To further investigate this possibility, we decided to look more closely at different GABAergic populations at different time points.

**Figure 2:**
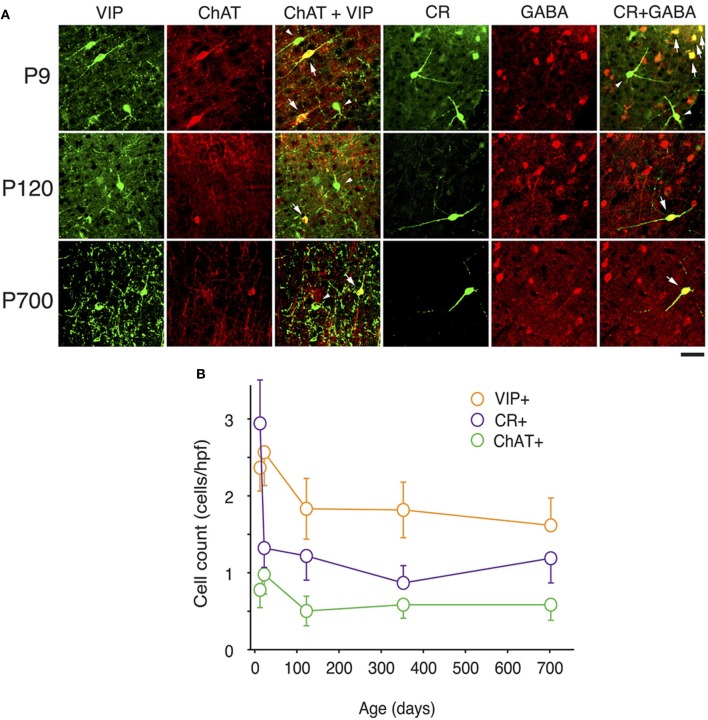
**Expression of CR, VIP, and ChAT markers in A1 GABAergic neurons across the lifespan. (A)** Representative high power (40X) confocal photomicrographs of ChAT+, VIP+, and CR+ immunolabeled cells for three age bins. Co-staining between VIP and ChAT was observed for 50% of neurons at all ages examined (arrows show cells with double labeling, triangles point to VIP+/ChAT− cells). Co-localization of CR and GABA was invariably observed except at P9 when 24% of CR+ neurons where GABA− (arrows show cells with double labeling, triangles point to CR+/GABA- cells). **(B)** VIP+, CR+, and ChAT+ immunolabeled cell counts averaged across all cortical layers over time (*n* = 28 animals with 21 photomicrographs per animal for each marker). Error bars are s.e.m. Scale bar: 50μm.

**Figure 3:**
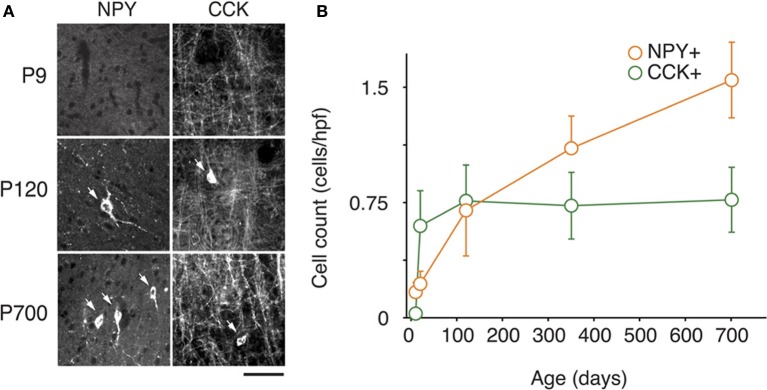
**Changes in NPY and CCK expression in A1 during life. (A)** Representative high power micrographs of NPY+ (left) and CCK+ immunolabeled cells (right) at three age intervals. The arrows mark the immunoreactive cells **(B)** NPY+ and CCK+ immunolabeled cell counts averaged across all cortical layers over time (*n* = 28 animals with 21 photomicrographs per animal for each marker). Error bars are s.e.m. Scale bar = 50μm.

**Figure 4:**
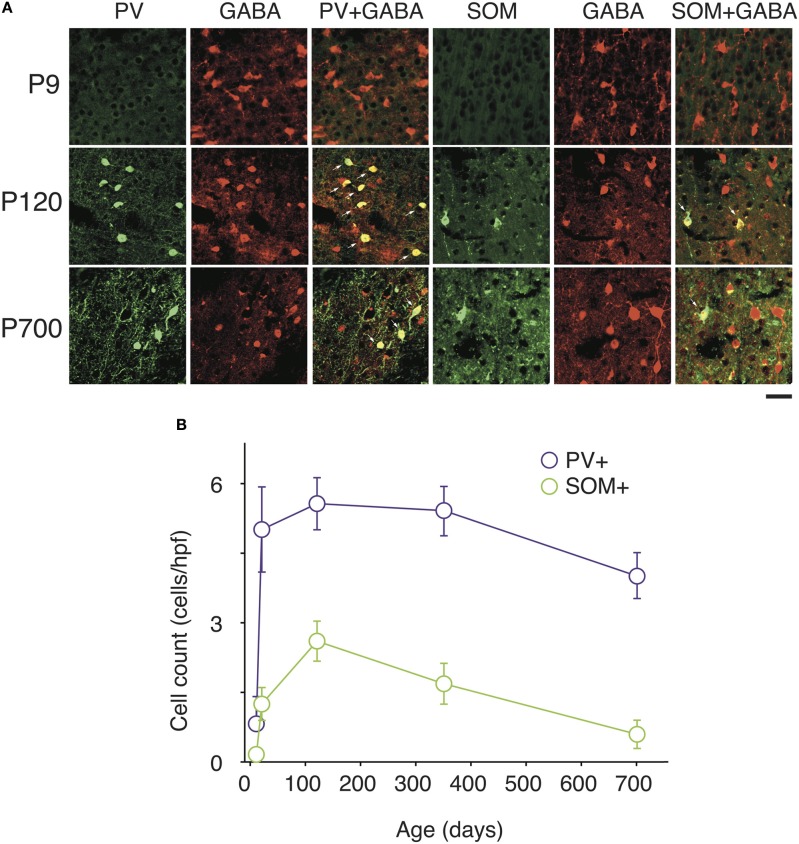
**Changes in PV and SOM markers expression in A1 GABAergic neurons with natural aging. (A)** Representative high power confocal photomicrographs of PV+ and SOM+ immunolabeled cells costained for GABA at each three age intervals. Note that all PV and SOM immunoreactive cells are also GABA+ (arrows) **(B)** PV+ and SOM+ immunolabeled cell counts averaged across all cortical layers over time (*n* = 28 animals with 21 photomicrographs per animal for each marker). Error bars are s.e.m. Scale bar = 50μm.

To study A1 at different ages, our samples were binned into 5 time points: immature cortex (P9), prior to critical period development and the development of functional inhibition in A1 (Chang et al., 2005; de Villers-Sidani and Merzenich, 2011), developing A1 postcritical period closure (P20), a young adult cortex (P120) with a plateau of interneurons population, older adult cortex point (P350) and aged cortex (P700–750). GABA co-labeling was also examined for all interneuron markers at all ages and except for one exception (see CR results below), all cells positive for one of the markers examined were also GABA immunoreactive (see Figures 1–4). Given this finding, GABA co-staining ratios are not presented in detail below.

**Figure 5:**
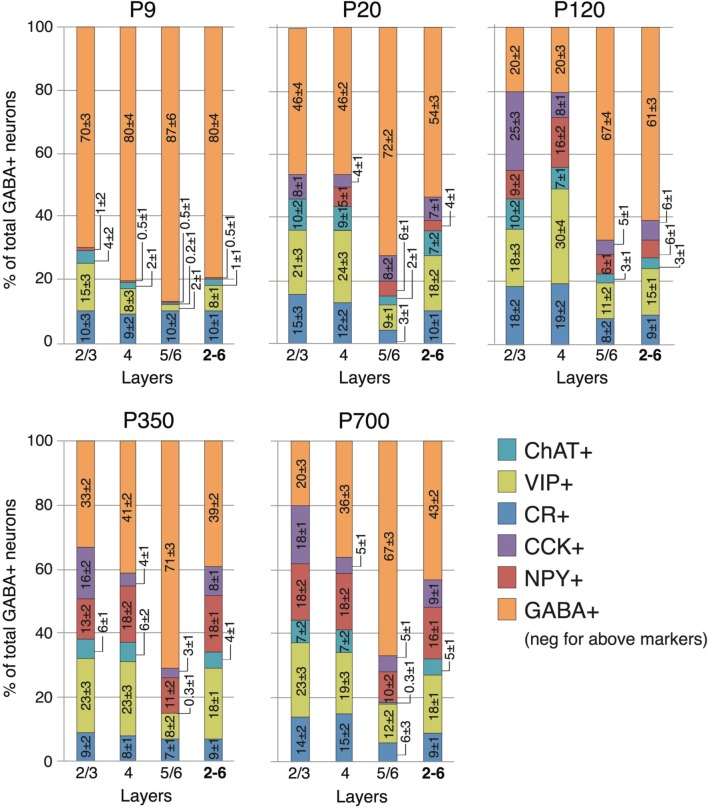
**Laminar distribution of CR, VIP, ChAT, NPY, and CCK neuron markers according to age**. Numbers represent the percentage of cells immunostained for the various interneuron markers relative to the total GABA+ cells count ± s.e.m. Orange GABA+ bars represent the percentage of cells immunoreactive for GABA but not showing co-staining for CR, VIP, ChAT, NPY, or CCK.

**Figure 6:**
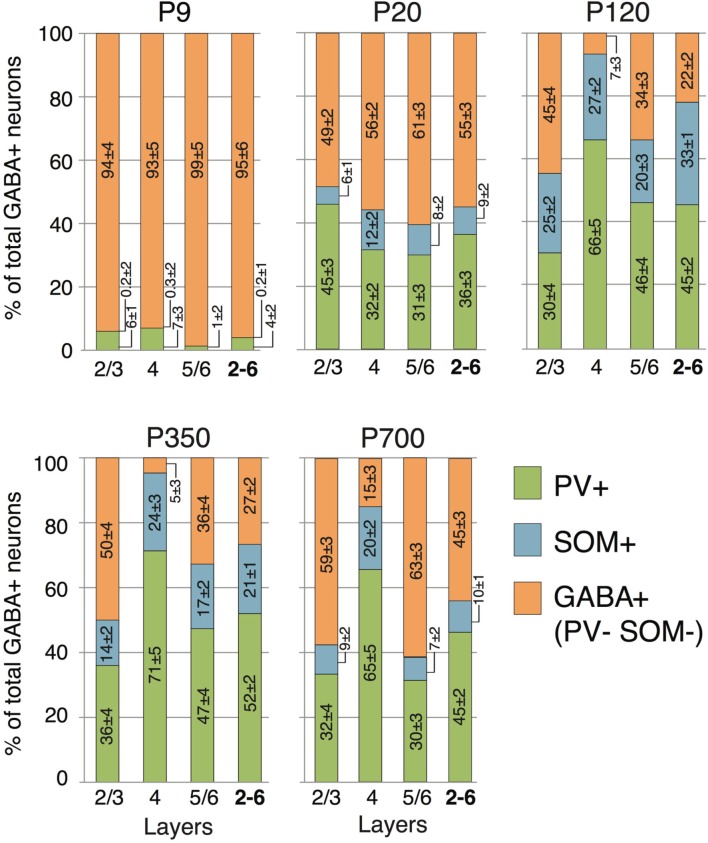
**Laminar distribution of PV and SOM neuron markers according to age**. Numbers reported in the columns represent the percentage of cells immunostained relative to the total GABA+ cells count ± s.e.m. Orange GABA+ bars represent the percentage of cells immunoreactive for GABA but not showing co-staining for either PV or SOM.

At P9, CR, VIP, and ChAT appeared to be the most prominent cortical interneuron markers; representing 10, 8, and 1% of total GABA positive cells (see Figure 2). This finding may be explained by delayed postnatal development of the most common markers in adults, PV, SOM, NPY and CCK, which were still minimally expressed in that second week of age. In previous studies focusing on the visual cortex, ChAT has been found to invariably co-localize to cells expressing VIP (Gonchar et al., 2008). We examined here if it was also the case in A1 and found that, at all ages, only ~50% of ChAT+ were also VIP+ (48 ± 6%; Figure 2). We also found that a subset CR+ cells at P9 were GABA− (24 ± 6%) whereas all of them were GABA+ at later ages. Between P9 and P20, we documented a significant 52% reduction in CR expression (2.9 ± 0.5 to 1.3 ± 0.3 cells/hpf; *p* = 0.008; Figure 2) and the appearance of CCK and SOM positive cells in A1 (CCK: from 0 to 0.57 ± 0.2 cells/hpf; *p* < 0.001; Figure 3; SOM: from 0.2 ± 0.03 to 1.4 ± 0.2 cells/hpf; *p* < 0.001; Figure 4). Interestingly, CR counts relative to the entire interneuron population were maintained despite the decrease in their absolute count in A1. This finding may be related to the 47% reduction in global GABA+ cells counts over the same time period. Another notable change between P9 and P20 was the significant 450% expansion of the PV cell population (*p* < 0.001; Figure 4). At P20, as seen in Figures 5, 6, PV+ cells became by far the dominant interneuron in A1 representing around 35% of the overall population followed by the CR (10%) and VIP (18%) populations.

Many interneurons had reached stable adult levels by P120 and showed minimal or no significant change in their marker expression from P20. That was the case for CCK, CR, ChAT, and PV (no change) and VIP (2.6 ± 0.3 to 1.8 ± 0.4 cells/hpf; *p* = 0.05). SOM+ cell counts however continued to increase and reached their lifetime maximum at around P120 (~100% increase to 2.75 ± 0.2; *p* = 0.01; Figure 4). NPY expression also steadily increased from P20 to P120 by more than three-fold (0.19 ± 0.04 to 0.74 ± 0.3 cells/hpf; *p* = 0.03; Figure 3). At this age the dominant interneuron population remained PV at 45% followed by SOM at 32% (see Figure 6). The relative population of the other interneuron markers changed only marginally during that period despite the significant 19% decrease in GABA+ cells between P20 and P120 (*p* = 0.002; Figure 1). These changes somewhat contrasted with what we observed in the P120–350 interval when the animals reached midlife. During that period we observed a substantial ~50% decrease in SOM expression (down to 1.5 ± 0.3 cells/hpf; *p* = 0.04; Figure 4) and a continued increase of NPY+ cells (up to 1.1 ± 0.3 cells/hpf; *p* = 0.04; Figure 3). By P350 SOM cells made up only 21% of the interneuron pool and NPY cells were almost as numerous at 18% of all interneurons (see Figures 5, 6). The total number of GABA+ cells also decreased by ~20% (12.1 ± 0.3 to 9.6 ± 0.2 cells/hpf; *p* = 0.02; Figure 1) during the same period, a change that cannot be fully explained the trajectories of SOM or NPY cells which are to few in absolute numbers to influence global GABA counts to this extent. As the animals grew into old age, the same trends continued including a further reduction in SOM+ cells (down to 0.9 ± 0.3 cells/hpf; *p* = 0.009; Figure 4), now not significantly different from their P20 value, and a further ~20% increase in NPY immunostaining (*p* = 0.04; Figure 3). During that later life period PV+ cell counts also declined significantly by 23% (*p* = 0.02; Figure 4). Total GABA+ and Nissl+ cell counts remained stable between P350 and P700, indicating against the changes in PV, SOM and NPY expression are to some extent independent from GABA staining fluctuations. It is important to note that all neurons taken together, these percentages add up to more than 100%, confirming that individual neurons express multiple markers and that the same neurons may have been were counted more than once for different markers (DeFelipe, 1993; Yan et al., 1995; Del Rio and DeFelipe, 1997; Kowiański et al., 2004; Xu et al., 2006; Gonchar et al., 2008). A summary of the different interneuron marker counts at all ages is found in Table 1.

**Table 1:** **Relative percentage of the interneuron markers examined in A1 at different ages**.

**Markers**	**Mean ± *SD* percent of GABAergic neurons in all layers**				
	**P9**	**P20**	**P120**	**P350**	**P700**
GABA	100	100	100	100	100
PV	4 ± 2	36 ± 3	45 ± 2	52 ± 2	45 ± 2
SOM	0.2 ± 1	9 ± 2	33 ± 1	21 ± 1	10 ± 1
CR	10 ± 1	10 ± 1	9 ± 1	9 ± 1	9 ± 1
VIP	8 ± 1	18 ± 2	15 ± 1	18 ± 1	18 ± 1
ChAT	1 ± 1	7 ± 2	3 ± 1	4 ± 1	5 ± 1
NPY	0.5 ± 1	4 ± 1	6 ± 1	18 ± 1	16 ± 1
CCK	0 ± 1	7 ± 1	6 ± 1	8 ± 1	9 ± 1

### Layer-based distribution of gabaergic inhibitory interneurons

Previous findings have suggested that interneuron origins, and rates of maturation, differentiation and specification can be correlated with laminar location (Anderson et al., 2002; Valcanis and Tan, 2003). Therefore, in order to create a detailed model of interneuron marker expression across the lifecourse, we quantified their presence in different layers. Figures 1, 5, 6 show how GABAergic interneuron populations were divided by layer, and how a proportion of these cells vary with time. Across all ages and layers, the largest overall population still appears to PV-expressing cells. SOM cells represented the second highest proportion of cells, but their numbers were largely dwarfed by their PV counterparts. Due to their proposed roles in cortical synchronization (Somogyi and Klausberger, 2005; Bartos et al., 2007), plasticity (Beierlein et al., 2000; Fries et al., 2002), and reported decline with aging (Kamal et al., 2013), we decided to examine these two groups of cells more closely.

We observed no SOM+ cells and only minimal staining for PV at P9, which was restricted to layers 2–4. The bulk of PV expression occurred as stated above between P9 and P20 with 2/3 of cells located in the thalamorecipient layer 4. SOM+ cells followed a very similar pattern and more than 50% were also located primarily in layer 4 at P9. For both of these cell types the main change between P20 and P120 was a significant increase in their populations in output layers 5/6 (PV: 50% increase; *p* = 0.01; SOM: 290% increase; *p* < 0.001; Figure 6). No significant change in PV counts was detected between P120 and P350, as expected from the global cell counts. As reported above, the total SOM population significantly declines between P120 and P350. This change is primarily the result of a 50% disappearance of these cells in superficial layers 2/3. No significant change in SOM was noted in the other layers during that period. Continuing to later life it was layers 5/6 that saw the greatest changes in both PV and SOM cells which were reduced by 32% (*p* = 0.02; Figure 6) and 60% (*p* = 006; Figure 6), respectively. At P350 both PV and SOM levels in layer 4 remain equivalent to P20–120 levels (*p* > 0.2).

Also in agreement with earlier studies in mouse (Gonchar et al., 2008), there were layer-based changes in cells expressing other neuropeptides (CR, VIP, ChAT). These three markers are all present at P9 and more prominently expressed in the more superficial cortical layers 2/3 and 4. This pattern is largely conserved throughout the life of the animal despite the proportional increase in each of those markers related to the loss of GABA+ non-otherwise marked cells during the P9-P20 period (Figure 5). CCK+ cells, which are first seen in A1 between P9 and P20, appear homogeneously over all cortical layers. Their population keeps increasing over the next 100 days but only in superficial layers 2/3 (310% increase; *p* < 0.001; Figure 5). Their counts then remain stable in all layers until P700. NPY expressing neurons, the only one that keep growing from birth to old age showed a similar overall layer specific pattern with earlier expression in deeper cortical layers. At P20, no NPY cells were found in layers 2/3. By P120, NPY expression reached a plateau in layers 4–6 but expression in layers 2/3 kept increasing up to P700 when they represented 18% of all interneurons in those layers (200% increase between P120 and P700; *p* = 0.01; Figure 5). Thus, it seems that both CCK and NPY+ cells, as well as those expressing PV and SOM have complex layer-based changes over the course of an animal's life and our earlier-predicted trajectory for the global population of GABAergic neurons in A1 cannot account for these. This limitation, as well as its possible implications will be further explored in the discussion section.

### Analysis of the trajectory of the different interneuron populations during life

**Figure 7:**
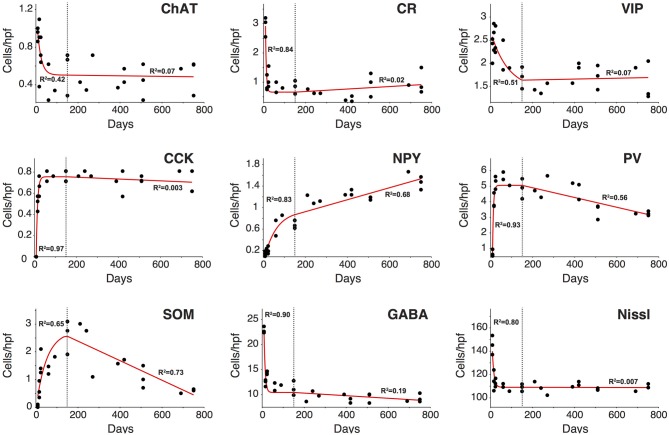
**Analysis of the trajectory of the different inhibitory cell markers during the life course**. Changes in all cell markers examined between P9 and P150 were best described by an inverse exponential decay or exponential decay function (red line). Most cell markers had reached a plateau by P150 after which point any observed change could be described by a linear function. Overall, only NPY, PV, and SOM displayed a significant change in count after P150 (dotted line). In all cases the change observed was gradual and started at around P150 (*n* = 28 animals with 21 photomicrographs per animal for each marker). R^2^ regression values are shown in each graph for the exponential function fit (left) and linear function fit (right).

As highlighted in the paragraphs above not all interneuron subtypes demonstrated comparable patterns of development and disappearance. To further characterize these trajectories we performed a regression analysis on each of them to determine what type of function would best describe their behavior during development adulthood and aging. Based on goodness of fit measures (Table 2), we first determined that the variation in interneuron markers could be reasonably well described by an exponential decay function in the first 150 days of life whereas a linear function was a better fit for P150–750. Based on these exponential functions we could then compute the time for each interneuron marker to reach their limiting value (asymptote) and time constant (T_1/2_) during development (Table 3). Figure 7 shows the data points and functions obtained for each interneuron marker. Overall we identified four distinct behaviors in the interneurons studied. Three types of interneurons (CR, VIP, and ChAT) displayed an exponential decay and had no significant change in their counts up to very old age. The rate of decay significantly differed between these 3 cell counts that stabilized at around P35, P90, and P140, respectively. All other counts (NPY, CCK, PV, and SOM) followed an inverse exponential decay during early development. PV+ and CCK+ cell counts both reached a plateau between 30 and 40 days of age whereas NPY+ and SOM+ ells stabilized later at around P145. In the P150–700 period CCK+ neuron counts remained very stable but both PV+ and SOM+ cells, as described previously, steadily decreased from P150 and after. Confirming our earlier results, the linear function fitted to NPY measurements is consistent with a progressive increase in their expression throughout the lifespan. Finally, we analyzed the variation in total A1 neuron (Nissl+) and interneuron populations (GABA+) using the same type of regression analysis. Both of these datasets followed an exponential decay similar to CR+ VIP+, and ChAT+ cells that stabilized at around P35-40. Nissl staining remained overall very stable over time and GABA+ showed a barely significant downward trend after P150. It should be noted that all cells positive for CR, VIP, ChAT, NPY, CCK, PV, and SOM were also GABA+ at all ages confirming their status as inhibitory interneurons. In conclusion, it appears that the majority of cortical interneuron changes after P150 were mediated by changing expression of PV−, SOM−, and NPY− expressing cells. Thus, with ours observations, we have established diverse patterns of GABAergic neurons development and disappearance over an animal's lifecourse.

**Table 2:** **Goodness of fit values for all functions describing the trajectory of the neural markers examined during development (P9–150) and aging (P150–750)**.

**Neuron markers**	**P9–150**					**P150–750**				
	**RMSE of exponential function**	***R*^2^**	***df***	***f*-value**	***P*-value**	**RMSE of linear function**	***R*^2^**	***df***	***f*-value**	***P*-value**
	****					****				
PV	0.4949	0.93	15	200	<0.0001	0.6030	0.56	15	19.9	0.0005
SOM	0.5634	0.65	15	28.4	0.0001	0.4782	0.73	15	40.6	<0.0001
CR	0.3448	0.84	15	122	<0.0001	0.3147	0.023	15	1.36	0.263
VIP	0.2917	0.51	15	17.6	0.0011	0.2732	0.065	15	0.09	0.769
ChAT	0.2166	0.42	15	11.2	0.0052	0.1656	0.070	15	0.02	0.890
NPY	0.1120	0.83	15	78.1	<0.0001	0.1653	0.68	15	33.6	<0.0001
CCK	0.0578	0.97	15	390	<0.0001	0.0758	0.0033	15	0.95	0.35
GABA	1.5437	0.90	15	118	<0.0001	1.0550	0.19	15	4.45	0.06
Nissl	6.9281	0.80	15	590	<0.0001	3.2404	0.0071	15	0.01	0.92

RMSE stands for root mean square error.

**Table 3:** **Summary of the main characteristics of all functions describing the trajectory of the neural markers examined**.

**Neurons subclasses**	**Time constant of exponential decay (t½; days ± 95% confidence intervals)**	**Time at 1% of asymptote value (days)**	**Rate of change of linear function (slope; cell per hpf/day ± 95% confidence intervals)**
**EXPONENTIAL DECAY AND PLATEAU**			
CR	4.05 ± 1.77	34	0.00043 ± 0.00078
VIP	82.3 ± 21.2	143	9.28e-5 ± 0.00041
ChAT	17.9 ± 4.26	91	−2.97e-5 ± 0.00041
**EXPONENTIAL DECAY FOLLOWED BY LINEAR DECREASE**			
PV	4.41 ± 1.56	30	−0.0031 ± 0.0015
SOM	56.1 ± 16.6	144	−0.0035 ± 0.0012
**INVERSE EXPONENTIAL DECAY AND PLATEAU**			
CCK	6.42 ± 3.21	40	−8.59e-5 ± 0.00019
**INVERSE EXPONENTIAL DECAY AND LINEAR INCREASE**			
NPY	59.3 ± 20.8	145	0.0011 ± 0.000041
**GLOBAL A1 CELL POPULATION**			
GABA	5.73 ± 1.96	38	−0.0026 ± 0.0027
Nissl	7.68 ± 2.36	36	−0.00042 ± 0.0081

## Discussion

Using immuonolabeling techniques, we have examined various populations of GABAergic interneurons in the rat auditory cortex across the animal's ordinary lifespan. In this study, neurons expressing PV, CR, SOM, VIP, NPY, CCK, and ChAT were monitored at 5 different time points, and across cortical layers. In addition to fluctuation in expression of many of our markers changed across the lifespan, it was found through Nissl staining that the total number neurons in the cortex appeared to decrease especially during early development. Findings by Burianova et al. (2009) have shown a robust age-related decrease in markers for GABAergic cells, suggesting that this decrease may be organized, in large part, by inhibitory interneurons. In agreement with this possibility, we have found numerous lines of evidence to suggest that programmed GABAergic cell death in a variety of interneuron populations death may, in fact, provide an integral part of cortical changes (thus, by extension, excitability) across the life course. The most notable instance of this was the robust decrease in CR, VIP and ChAT cell markers before P20. While seemingly a large and indiscriminate cell death programmed, this time point, in fact coincides with the beginning of mature sounds representation in A1 (de Villers-Sidani et al., 2008). This tightly controlled process, relying on a tight balance of excitation and inhibition is essential for mature cortical function, and depends heavily on both environmental input and neurochemical environment (de Villers-Sidani and Merzenich, 2011; Anomal et al., 2013). However, this type of decline was not global, as PV and SOM-expressing neurons increased in a parabolic curve until late adulthood, when they, too, began to decline. In contrast, CR, VIP, and CCK-expressing cells maintained relatively stable expression across adulthood, with the above-mentioned early decrease in CR, VIP, and ChAT, and concomitant increase in NPY and CCK. Taken together, these trends suggest an orderly, programmed population trajectory that may be easily modeled through exponential decay curves followed by linear functions.

One of the most interesting aspects of our findings comes from the paucity of NPY, CCK, SOM, and PV expression early in the lifecourse (P20–120). To reiterate, our study was not able to determine whether this reflects evidence of low cell populations *per se*, but may rather reflect differing protein elaboration. While the latter possibility has not been fully explored, previous work in rodents, monkeys and humans has shown that these inhibitory interneuron markers have sparse expression in early developing cortex (Hof et al., 1999; Giacobini and Wray, 2008; Zecevic et al., 2011), but few have followed their trajectories over the entire lifecourse. What role could interneurons expressing these markers play in development? NPY is perhaps best known for its role in feeding, anxiety, and other homeostatic behaviors (Lin et al., 2004), but changing levels of its receptor expression have been implicated in age-related memory deficits and Alzheimer's disease (Minthon et al., 1996; Borbély et al., 2013). Furthermore, its tight coupling with cholinergic systems in the brain has pointed toward communication with other neuron subtypes in the cortex. CCK, too, is closely involved with a host of developmental processes in early life, including learning and sexual maturation (Giacobini and Wray, 2008). While all of these neuropeptide markers underlie a rich variety of cortical functions, our work has found them (with the exceptions of CCK) to be united by a common developmental trajectory, starting with low levels and plateauing around P120. This provides a large time window for functional maturation and opens exciting possibilities for exploring functional consequences of these fluctuating markers early in development.

In later life, this work has also provided important insights about the changing nature of GABAergic transmission. Unlike other markers, both PV and SOM showed a significant decrease beginning in mid-life between P120 and P350 and continuing to late adulthood at P700. It is unclear what special traits these markers may possess that somehow single them out for age-related population decline, but previous work has shed a great deal of light on their role in cognitive and sensory impairment. As early as 1993, both PV and SOM were known to decrease in sensory cortices (Miettinen et al., 1993), and since then, many studies have replicated these findings in other species and regions of the brain (Ouda et al., 2008; Caballero et al., 2013; Fish et al., 2013). For example, several groups have shown that SOM appears to be especially sensitive to both injury and aging than other interneuron subtypes (Lowenstein et al., 1992; Stanley et al., 2012). Furthermore, SOM's role in sensory processing and hippocampal excitation have implicated in several neurodegenerative conditions including Alzheimer's and Parkinson's disease (Patel, 1999; Viollet et al., 2008; Martel et al., 2012; Lin and Sibille, 2013). PV also seems to play a special role in age-related A1 functional decline (Ouda et al., 2008; Del Campo et al., 2012), however differs from SOM in one key aspect: its ability to be modified in response to activity in later life. Both de Villers-Sidani et al. (2010) and Kamal et al. (2013) have shown that PV-containing cells are sensitive to environments carrying high and low auditory information, respectively, and can up- or down-regulate their characteristic protein expression accordingly. This work raises the possibility that the expression of markers in cortical GABAergic neurons may be more sensitive to environmental information than previously believed. Thus, population fluctuations may better represent external stimuli than an ongoing neurodegenerative process.

However, PV and SOM were not the only cell types to show dramatic change in later life. Surprisingly and unexpectedly given previous findings from humans (Cha et al., 1996, 1997), NPY showed a significant increase between P20 and P350. We cannot fully explain this result, but offer a few tentative hypotheses: first, in humans, reduced NPY metabolism has been strongly correlated with Alzheimer's disease (Minthon et al., 1996; Borbély et al., 2013), suggesting that if NPY up-regulation is a consequence of normal aging, then its failure could lead to pathological results. Alternatively, NPY's involvement in cholinergic transmission (Nakhate et al., 2009) may be implicated, as the density of ChAT positive processes in our animals were greatly reduced in old age despite the stability of that cell population. NPY-expressing cells might thus have undertaken a compensatory mechanism for their ChAT counterparts, or more broadly for decay of PV and SOM expression. Our finding can support this interpretation that, despite widespread disappearance of the two most prominent GABAergic interneuron types, PV and SOM, numbers of cortical GABAergic cells remained essentially unchanged after P350.

In terms of global cortical changes over development and aging, our work in the present study has unearthed two major patterns. Early in life, all neuron subtypes were subject to exponential change, a pattern closely tied to the critical period window (de Villers-Sidani et al., 2007, 2008). However, in PV-, SOM-, and NPY-expressing cells change from P150 to P750 takes on a form more consistent with linear change. The remainder of our markers, CCK, ChAT, VIP, and CR, show relatively stable populations throughout adult life. At the most simple level, changes in interneuron marker expression may be grouped into four distinct categories: inverse exponential decay and plateau (CCK), inverse exponential decay and linear increase (NPY), exponential decay and plateau (ChAT, VIP, and CR), and inverse exponential decay followed by linear decrease (PV and SOM). Could these categories possibly be reflected by functional cortical characteristics? While answers remain elusive for the first three groups, there is good evidence to suggest that PV and SOM disappearance correlated temporally with the emergence of auditory cognitive impairments (Ouda et al., 2008; Burianova et al., 2009; de Villers-Sidani et al., 2010). Rather than pure cell death, these results could also reflect a large down-regulation of protein expression, as evidenced by resurgence of PV in Kamal et al. (2013). Whatever the cause, our work has provided a careful, population-based framework for monitoring different subtypes of GABAergic neurons and provided comparative population trends over the lifecourse.

Despite its successes, our study is, however, limited in several respects. First, our observations were limited to A1, which, although mirroring structural and functional aspects of many other cortical areas cannot accurately speak to changes in other brain regions. Secondly, our study was conducted in single strain of rats living in a relatively simple laboratory environment. Previous work has shown that both strain (Ouda et al., 2008) and environmental enrichment (de Villers-Sidani et al., 2010) can have a significant effect on interneuron populations. Additionally, our study did not systematically explore all possible interneuron markers, excluding, for example calbindin, NOS, doublecortin and 5-HT3. In the future, perhaps these interneuron subtypes may be fruitfully explored, and provide insights on the nature of GABAergic signaling and aging. It should also be mentioned that GABA immunoreactivity may provide only an estimate of GABAergic population as some inhibitory neurons might in some circumstance not be revealed by such antibodies. For example Von Engelhardt et al. (2007) found that only a subset of ChAT positive neurons co-express GAD67. In this study we have also noted that at P9 25% of CR+ neurons were GABA negative. In future work with more advanced co-labeling techniques, perhaps these challenges may be resolved, providing the most accurate picture possible of how GABAergic interneurons change across the lifecourse.

In conclusion, this work has shown that different GABAergic interneuron subtypes display remarkable diversity across an animal's life, and coincide with many important functional benchmarks in development and aging. At first undergoing exponential increase, NPY, CCK-expressing cells plateau relatively early; while PV and SOM-expressing cells later show a marked decrease. ChAT, VIP, and CR-expressing cells exhibit an opposite pattern, undergoing a rapid decrease in just the first few days of life. These results shed light on the already complex debate surrounding inhibition and aging, and provide some insights on just how the cortex's profile of excitation and inhibition changes across the lifecourse. With respect to the reduced inhibition seen in the aged auditory system (Caspary et al., 2008; de Villers-Sidani et al., 2010), we believe that our characterization of PV and SOM-expressing cells' decline represents a fruitful avenue for future research. Perhaps understanding the delicate balance of inhibitory transmission in normal aging will provide clues to ongoing functional processes or even neurodegenerative processes such as Alzheimer's disease. Whatever the outcome, we believe that this characterization of cortical GABAergic interneurons has provided intriguing questions for future research, and helped further enrich our image of the brain from birth to death.

## Author contributions

Lydia Ouellet and Etienne de Villers-Sidani designed the experiments. Lydia Ouellet performed the experiments. Etienne de Villers-Sidani and Lydia Ouellet performed analysis. Lydia Ouellet and Etienne de Villers-Sidani wrote the paper.

### Conflict of interest statement

The authors declare that the research was conducted in the absence of any commercial or financial relationships that could be construed as a potential conflict of interest.
